# Integrating Hyperspectral Data and Deep Learning for Non-Destructive Prediction of Tea Quality Parameters Across Different Physical States of Tea Leaves and Growth Periods

**DOI:** 10.3390/plants15071071

**Published:** 2026-03-31

**Authors:** Guanzi Zhou, Haotian Ji, Rongyu Pan, Xiaowei Yang, Suhui Zhao, Lei Yang, Xiaohan Shang, Huijie Zhang, Hanchi Zhang, Xiaojun Liu, Yuanchun Ma, Xujun Zhu, Jie Jiang, Wanping Fang

**Affiliations:** 1 College of Horticulture, Nanjing Agricultural University, Nanjing 210095, China; 2 Guizhou Tea Research Institute, Guizhou Academy of Agricultural Sciences, Guiyang 550006, China; 3 National Engineering and Technology Center for Information Agriculture, MOE Engineering Research Center of Smart Agricultural, MARA Key Laboratory for Crop System Analysis and Decision Making, Jiangsu Key Laboratory for Information Agriculture, Institute of Smart Agriculture, Nanjing Agricultural University, Nanjing 210095, China; 4 College of Rural Revitalization, Jiangsu Open University, Nanjing 214257, China

**Keywords:** tea quality, hyperspectral data, multi-domain spectral features, deep learning, non-destructive estimation

## Abstract

Achieving rapid and non-destructive assessment of tea quality is essential for intelligent tea production and quality control. In this study, an integrated hyperspectral and deep learning framework was developed to estimate tea quality constituents across seasons and physical states. Samples included field fresh leaves, dried tea leaves, and tea powder, were collected in spring, summer, and autumn. Tea polyphenols and catechins were predicted using original reflectance, harmonic features, and wavelet features fused into multi-domain indices. Extreme gradient boosting, Gaussian process regression, and convolutional neural networks (CNN) were systematically compared to construct the quality estimation models. The result showed that three-feature indices consistently outperformed two-feature indices, yielding R^2^ from 0.48 to 0.71. CNN achieved the best overall performance among the three modeling approaches, with its optimal accuracy obtained for tea powder samples in autumn, yielding R^2^ values of 0.81 and 0.76 for tea polyphenols and catechins, respectively. This framework provides an accurate, non-destructive tool for tea quality evaluation and traceability, offering technical support for intelligent agriculture and quality control across the tea industry chain.

## 1. Introduction

As one of the most widely consumed natural beverages worldwide, tea quality not only determines its sensory attributes, such as taste and aroma, but is also closely associated with a range of health benefits, including antioxidant, anti-inflammatory, lipid-lowering, and cardiovascular protective effects. Among the numerous bioactive compounds present in tea, tea polyphenols are widely recognized as the dominant biochemical constituents governing both functional properties and sensory quality. Owing to their strong antioxidant capacity, free radical-scavenging activity, and ability to mitigate oxidative stress, tea polyphenols play a critical role in reducing the risk of chronic diseases and promoting human health. From a sensory perspective, they contribute directly to bitterness, astringency, color formation, and overall flavor balance, while from a processing perspective they serve as key precursors for oxidation products such as theaflavins and thearubigins that determine the quality of fermented teas. Consequently, tea polyphenols are widely regarded as primary quality markers and functional value determinants in tea evaluation systems. Together with caffeine and free amino acids, they constitute core indicators for assessing tea quality and market grade [1]. With the ongoing transition of the tea industry toward high-quality, digitalized, and intelligent production systems, the development of rapid, non-destructive, and real-time monitoring technologies for these critical components—particularly tea polyphenols as the principal quality determinant—has become an urgent and fundamental requirement for modern tea production.

In recent years, non-destructive sensing techniques such as fluorescence spectroscopy, near-infrared spectroscopy (NIR), and hyperspectral imaging have provided new opportunities for quantitative and real-time monitoring of tea quality [2]. Among the various biochemical constituents determining tea quality, tea polyphenols and catechins are considered the most critical indicators because they strongly influence tea flavor, astringency, antioxidant capacity, and overall commercial value. To accurately quantify these key compounds, hyperspectral and spectroscopic techniques have been increasingly applied. Wang et al. [3] and Luo et al. [4] demonstrated that hyperspectral imaging, combined with chemometric methods, effectively captures variations in tea polyphenol content, enabling reliable non-destructive prediction and revealing strong spectral–polyphenol correlations across different tea types. While Liu et al. [5] and Luo et al. [6] applied visible and near-infrared spectroscopy combined with feature selection to achieve non-destructive determination of multiple tea polyphenols and simultaneous quantification of major catechins in fresh and black tea, demonstrating that spectral techniques can efficiently capture key quality compounds and support rapid, reliable tea quality evaluation. Recent studies have also explored the integration of spectral sensing technologies with advanced machine learning algorithms to further improve prediction accuracy. For instance, Yang et al. combined Fourier transform near-infrared spectroscopy with machine learning methods to simultaneously predict tea polyphenols and epigallocatechin gallate (EGCG), one of the most important catechin components contributing to tea quality and health-related functionality [7]. These advances highlight the strong potential of hyperspectral and near-infrared spectroscopy, especially when integrated with chemometrics and machine learning approaches, for accurate prediction of tea polyphenols and catechins. Collectively, establishing a unified non-destructive monitoring framework capable of accurately predicting tea polyphenols and catechins across different stages—from fresh leaves to processed tea products—would significantly improve the understanding of quality formation and transformation mechanisms. Such approaches would also promote intelligent quality evaluation and end-to-end management throughout the tea production chain.

At present, most hyperspectral sensor-based studies on quality prediction still focus primarily on the use of original reflectance spectra or conventional spectral indices. For instance, Kang et al. established correlations between hyperspectral reflectance in the 400-1000 nm range and catechins components (EC, EGC, CG, and EGCG), with coefficients of determination (R^2^) ranging from 0.42 to 0.88 [8]. Similarly, Chen et al. identified the normalized difference vegetation index and the wide dynamic range vegetation index as the optimal indices for predicting polyphenols, sugars, amino acids, and caffeine [9]. However, most of these studies rely on raw spectra or a single preprocessing strategy and fail to fully exploit the complementary information contained in different spectral feature domains. In fact, different feature extraction methods emphasize distinct aspects of spectral information. Original reflectance spectra directly characterize the overall leaf reflectance behavior and serve as the fundamental basis for subsequent analysis [10,11]. Continuous wavelet transform (CWT) is effective in capturing local absorption features and subtle chemical variations, demonstrating advantages in stress detection and category discrimination [12,13]. Harmonic analysis, on the other hand, separates low-frequency trends from high-frequency details in spectral signals, thereby helping to suppress baseline drift and fluctuations caused by illumination variability [14]. Nevertheless, existing research has rarely integrated these three forms of hyperspectral features for the joint prediction of tea quality indicators, particularly tea polyphenols content, which represents the most critical biochemical determinant of tea functional quality and economic value.

In terms of modeling strategies, conventional regression models often exhibit limited predictive performance when dealing with the high dimensionality, strong multicollinearity, and complex nonlinear relationships inherent in hyperspectral data. In recent years, machine learning approaches have demonstrated strong feature learning capability and model generalization, enabling effective performance in complex prediction tasks [15,16]. Algorithms such as extreme gradient boosting (XGBoost) and Gaussian process regression (GPR) are capable of capturing complex nonlinear structures in hyperspectral data while offering advantages in feature selection and uncertainty quantification. In particular, deep learning algorithms, represented by convolutional neural networks (CNN), can automatically learn local spectral patterns and deep nonlinear representations. These methods have shown outstanding performance in plant nutrition and quality prediction. For example, Luo et al. employed one-dimensional and two-dimensional CNN to extract deep spectral and spatial features from hyperspectral images of tea leaves and developed non-destructive prediction models for tea polyphenol content [17]. Their results demonstrated that models based on combined spectral–spatial deep features achieved a coefficient of determination (R^2^) of approximately 0.94 on the test set. In addition, deep learning approaches have demonstrated significant advantages in tea planting density estimation, yield prediction, and the retrieval of physiological and biochemical parameters [18,19]. Consequently, developing a deep learning-based multi-source feature fusion framework represents a promising strategy for improving the accuracy and robustness of tea quality estimation.

This study aims to establish a unified, non-destructive framework for tea quality prediction by integrating multi-domain spectral features with deep learning. By incorporating original reflectance, harmonic, and wavelet components across multiple seasons and physical states, the framework captures complementary spectral information, enabling robust, real-time monitoring and precision regulation of tea polyphenols. Compared with prior studies that relied primarily on raw reflectance or single preprocessing techniques, this approach provides a more comprehensive and reliable foundation for intelligent quality control and value-oriented management across the tea industry chain. The specific research objectives are detailed as follows: (1) to extract original reflectance spectra (R), harmonic features (HF), and wavelet features (WF) from tea samples in three physical states (field fresh leaves, dried tea leaves, and tea powder), and to construct both dual-feature and triple-feature spectral indices sensitive to quality-related constituents, with particular emphasis on tea polyphenols as the principal evaluation target; (2) to develop prediction models for tea quality components using Gaussian process regression (GPR), extreme gradient boosting (XGBoost), and convolutional neural networks (CNN) based on the selected optimal spectral indices, and to systematically compare their predictive performance across different tea physical states; and (3) to evaluate the applicability and robustness of the developed models across spring, summer, and autumn seasons, and to elucidate, from a spectroscopic perspective, the mechanisms by which seasonal metabolic variations influence model performance.

## 2. Results

### 2.1. Seasonal Variation Characteristics of Tea Quality Components

**Figure 1:**
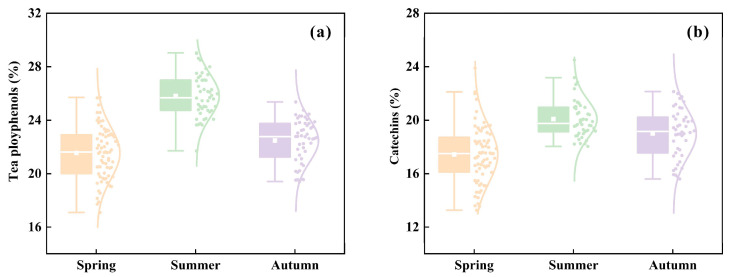
Seasonal variations in tea quality components: (**a**) tea polyphenols; (**b**) catechins.

Chemical analyses of tea samples collected in spring, summer, and autumn revealed significant seasonal differences in both tea polyphenol and catechin contents (Table 1). One-way analysis of variance (ANOVA) showed that season had a highly significant effect on tea polyphenol content (F(2,165) = 88.065, *p* < 0.001), as well as on catechin content (F(2,161) = 29.358, *p* < 0.001). Post hoc analysis using the Tukey HSD test further clarified the seasonal variation patterns of these two quality indicators. Tea polyphenol content was highest in summer (25.82% ± 1.97%), which was significantly higher than that in autumn (22.48% ± 1.62%) and spring (21.54% ± 1.94%) (*p* < 0.05), while autumn was significantly higher than spring (*p* = 0.013). A similar pattern was observed for catechin content, with summer (20.08% ± 1.42%) being significantly higher than autumn (19.00% ± 1.83%) and spring (17.43% ± 2.13%) (*p* < 0.05), and autumn significantly higher than spring (*p* < 0.001). Overall, both tea polyphenol and catechin contents exhibited a decreasing trend from summer to autumn and further to spring(Figure 1).

### 2.2. Analysis of Hyperspectral Reflectance Under Three Physical States

**Figure 2:**
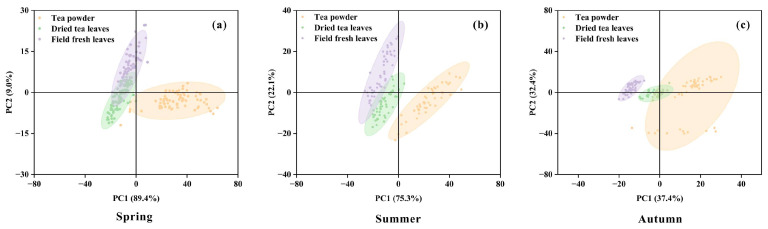
Principal component analysis (PCA) score plots of hyperspectral reflectance data of tea leaves under different physical states in (**a**) spring, (**b**) summer, and (**c**) autumn.

PCA was applied to the hyperspectral data of three seasons and three physical states (field fresh leaves, dried tea leaves, and tea powder) for dimensionality reduction and visualization, revealing the combined effects of physical state and season on spectral characteristics (Figure 2). The results showed distinct clustering patterns across seasons. However, substantial differences were observed in both the variance explained by the principal components and the degree of class separation. In spring, the first principal component (PC1) accounted for 89.4% of the total variance, providing the highest discriminative power. In summer, the contribution of PC1 decreased to 75.3%, and partial overlap between field fresh leaves and dried tea leaves clusters was observed. By autumn, the spectral information became more dispersed, with the first two principal components contributing more evenly to the total variance (37.4% and 32.4%, respectively). Overall, in spring and summer, powdered samples exhibited distinct spectral characteristics and were clearly separable from field fresh leaves and dried tea leaves, whereas in autumn, discrimination became relatively more challenging.

### 2.3. Construction of Sensitive Hyperspectral Features and Hyperspectral Indices

**Figure 3:**
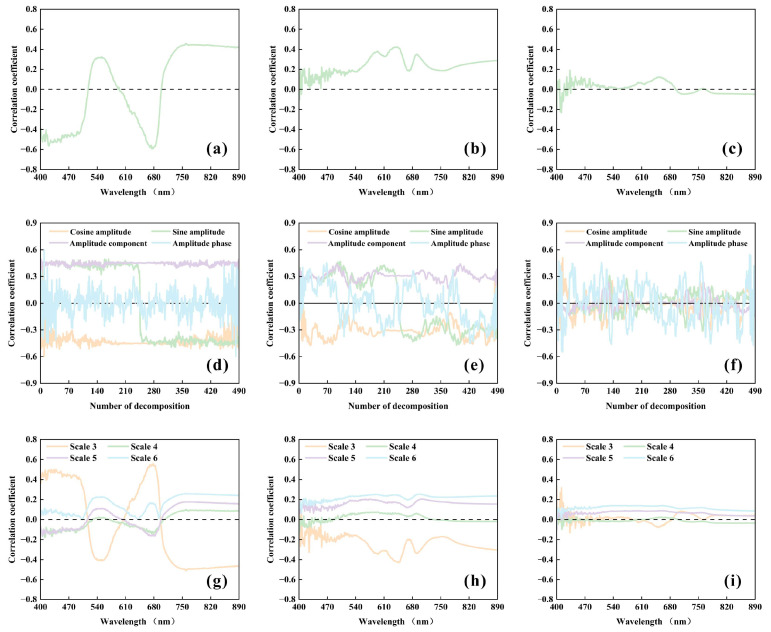
Quantitative correlations between different spectral features and tea quality indicators (tea polyphenols used as an example) for spring tea samples under different physical states: Panels (**a**–**c**) show the correlations between raw reflectance spectra (R) and tea polyphenols for field fresh leaves (**a**), dried tea leaves (**b**), and tea powder (**c**). Panels (**d**–**f**) show the correlations between frequency-domain features (HF) and tea polyphenols for field fresh leaves (**d**), dried tea leaves (**e**), and tea powder (**f**). Panels (**g**–**i**) show the correlations between wavelet features (WF) and tea polyphenols for field fresh leaves (**g**), dried tea leaves (**h**), and tea powder (**i**).

To investigate the contribution of information from different feature domains to quality prediction, correlations between quality indicators (using spring tea polyphenols as an example) and three types of spectral features (R, HF, and WF) were first analyzed (Figure 3). In the harmonic analysis, the original reflectance curves within the 400–890 nm range were decomposed into CA, SA, A, and P parameters at different harmonic orders. Wavelet analysis was performed by multi-scale decomposition (S = 3 to 6) to extract local fluctuation coefficients of the original reflectance at each scale (WF). The results showed that, in the original reflectance spectra, field fresh leaves exhibited the most pronounced negative correlation extremum at 675 nm (R = 0.59), indicating that this band is highly sensitive to variations in tea polyphenol content. Meanwhile, a stable positive correlation region was observed in the near-infrared region around 750 nm. Dried tea leaves samples generally showed positive correlations, with the highest sensitivity occurring at 637 nm (R = 0.42), whereas correlations in powdered samples were markedly reduced. Compared with raw reflectance, harmonic decomposition substantially enhanced the spectral–quality relationships. Among the HF, CA emerged as the dominant component, exhibiting the strongest correlation in field fresh leaves (R = 0.60). This enhancement effect was reduced in dried tea leaves and was relatively limited in powdered samples. In the wavelet domain, both field fresh leaves and dried tea leaves samples showed higher correlations with tea polyphenols predominantly at the S3 scale, with sensitive bands concentrated around 678 nm (R = 0.56) and 645 nm (R = 0.43), respectively. In contrast, powdered samples exhibited consistently weak correlations across all scales. Overall, these results demonstrate that multi-domain feature extraction effectively enhances the representation of key information associated with tea quality, while the magnitude of this enhancement is strongly influenced by the physical state of the samples.

**Figure 4:**
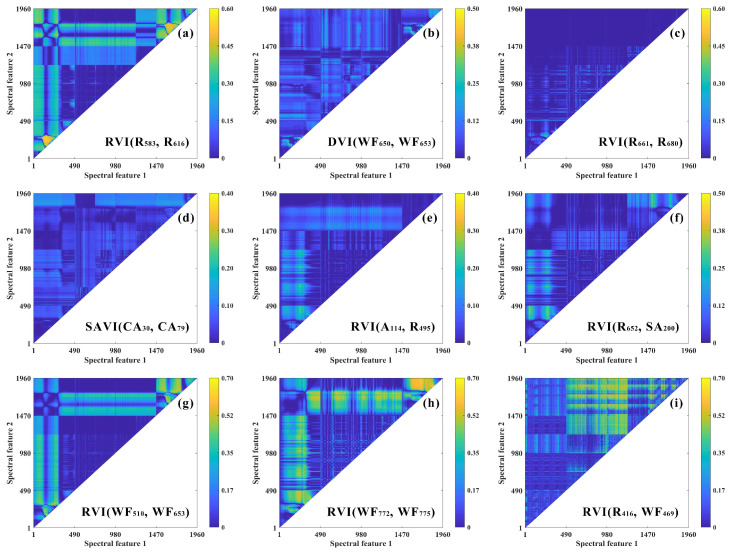
Comparison of the predictive performance of hyperspectral two-feature indices for major tea quality parameters (tea polyphenols used as an example) under different seasonal conditions, evaluated by R^2^ across different physical states: Panels (**a**–**c**) show the prediction results for spring for field fresh leaves (**a**), dried tea leaves (**b**), and tea powder (**c**). Panels (**d**–**f**) show the prediction results for summer for field fresh leaves (**d**), dried tea leaves (**e**), and tea powder (**f**). Panels (**g**–**i**) show the prediction results for autumn for field fresh leaves (**g**), dried tea leaves (**h**), and tea powder (**i**). Note: In the coordinate system, bands 1–490 correspond to R, 490–1470 correspond to HF, and 1470–1960 correspond to WF.

**Figure 5:**
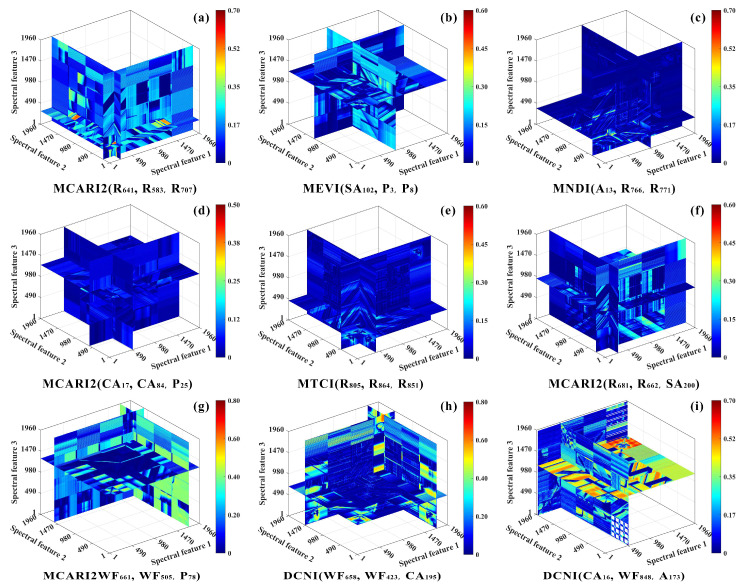
Comparison of the predictive performance of hyperspectral three-feature indices for major tea quality parameters (tea polyphenols used as an example) under different seasonal conditions, evaluated by R^2^ across different physical states: Panels (**a**–**c**) show the prediction results for spring for field fresh leaves (**a**), dried tea leaves (**b**), and tea powder (**c**). Panels (**d**–**f**) show the prediction results for summer for field fresh leaves (**d**), dried tea leaves (**e**), and tea powder (**f**). Panels (**g**–**i**) show the prediction results for autumn for field fresh leaves (**g**), dried tea leaves (**h**), and tea powder (**i**). Note: In the coordinate system, bands 1–490 correspond to R, 490–1470 correspond to HF, and 1470–1960 correspond to WF.

Based on these results, an exhaustive search algorithm was applied to systematically construct and screen optimal two-feature and three-feature hyperspectral indices using the aforementioned band index formulations (Table 2). Using spring as a representative season, the screening results indicated that three-feature indices generally achieved higher predictive performance than two-feature indices. Specifically, R^2^ ranged from 0.48 to 0.65 for three-feature indices and from 0.35 to 0.58 for two-feature indices. (Figure 4 and Figure 5). In addition, the constituent features of the optimal indices varied markedly with physical state. For field fresh leaves, the optimal indices were predominantly composed of band combinations from the original reflectance spectra in the red to red-edge regions, such as RVI(R_583_, R_616_) (R^2^ = 0.58) and MCARI2(R_641_, R_583_, R_707_) (R^2^ = 0.65). In the dried tea leaves state, combinations involving harmonic-domain and wavelet-domain features occurred more frequently among the optimal indices, including MEVI(SA_102_, P_3_, P_8_) for tea polyphenols (R^2^ = 0.54) and MEVI(SA_130_, SA_179_, WF_708_) for catechins (R^2^ = 0.48). In contrast, in powdered samples, higher-order components associated with the harmonic domain became dominant, as exemplified by MNDI(A_13_, R_766_, R_771_) for tea polyphenols (R^2^ = 0.63) and MTCI(R_736_, R_763_, R_755_) for catechins (R^2^ = 0.49). These findings suggest that, for different physical states of tea samples, the most sensitive hyperspectral indices should be identified from distinct feature domains.

### 2.4. Development of Non-Destructive Prediction Models for Tea Quality Indicators Based on Deep Learning Algorithms

After systematically comparing the modeling performance of XGBoost, GPR, and CNN, the CNN model was found to exhibit overall superior performance in terms of prediction accuracy and stability, demonstrating the best overall predictive performance among the three approaches (Table 3). The results further revealed a pronounced seasonal dependency in the predictive accuracy of hyperspectral models for tea quality components. For the same physical state, model performance generally followed the order of autumn > summer > spring. For example, in predicting catechins in powdered samples, the model achieved higher accuracy in autumn (R^2^ = 0.76, RMSE = 0.89%, RE = 5.03%) than in spring (R^2^ = 0.75, RMSE = 1.07%, RE = 6.42%), with both improved R^2^ and lower errors in autumn. In addition, as samples transitioned from field fresh leaves to dried tea leaves through dehydration processing, and further to powdered form via grinding, model performance exhibited a stepwise improvement. Overall, prediction accuracy exhibited a consistent pattern, whereby tea powder and dried tea leaves showed higher prediction accuracy than field fresh leaves. Taking catechins in spring as an example, the powdered-sample model achieved an R^2^ (0.75) comparable to that of dried tea leaves (0.75) and higher than that of field fresh leaves (0.72), while yielding the lowest RMSE (1.07%) and RE (6.42%) among the three physical states in spring. Moreover, the prediction difficulty varied among different chemical components. In autumn tea powder samples, the predictive models for tea polyphenols were the most accurate and robust, achieving an R^2^ of 0.81 with the smallest errors (RMSE = 0.70%, RE = 3.11%), while catechins also showed strong performance (R^2^ = 0.76, RMSE = 0.89%, RE = 5.03%). Overall, the CNN model achieved the best predictive performance for tea polyphenols and catechins in autumn tea powder samples, with R^2^ values of 0.81 and 0.76, respectively, demonstrating its strong applicability in hyperspectral-based quality modeling.

### 2.5. Model Reproducibility of Seasonal and Physical State Differences

One-way ANOVA followed by Tukey HSD post hoc tests were performed on the CNN-predicted tea polyphenols and catechins contents across different seasons and physical states (Table 4 and Table 5). For tea polyphenols, significant seasonal differences were observed in field fresh leaves (F = 124.90, *p* < 0.001), dried tea leaves (F = 140.03, *p* < 0.001), and tea powder (F = 113.72, *p* < 0.001), with all post hoc comparisons showing summer (25.83%, 25.79%, 25.80%) significantly higher than autumn (22.52%, 22.50%, 22.44%) and spring (21.54%, 21.45%, 21.55%). For catechins, significant seasonal differences were also found in field fresh leaves (F = 37.12, *p* < 0.001), dried tea leaves (F = 40.35, *p* < 0.001), and tea powder (F = 46.50, *p* < 0.001), with summer (20.01%, 20.11%, 20.16%) significantly exceeding autumn (19.00%, 19.11%, 19.04%) and spring (17.51%, 17.45%, 17.49%). In contrast, no significant differences were detected among the three physical states within any season for either tea polyphenols (spring: F = 0.084, *p* = 0.919; summer: F = 0.016, *p* = 0.984; autumn: F = 0.046, *p* = 0.955) or catechins (spring: F = 0.028, *p* = 0.973; summer: F = 0.169, *p* = 0.845; autumn: F = 0.065, *p* = 0.937). These results establish that seasonal variation significantly affects tea quality components with a consistent summer > autumn > spring pattern, while physical states do not significantly alter the intrinsic chemical composition. These statistical patterns serve as the benchmark for evaluating model performance. These results confirm that the CNN model successfully preserves the intrinsic statistical structure of the original measurements.

### 2.6. Feature Importance Analysis of CNN Predictions

**Figure 6:**
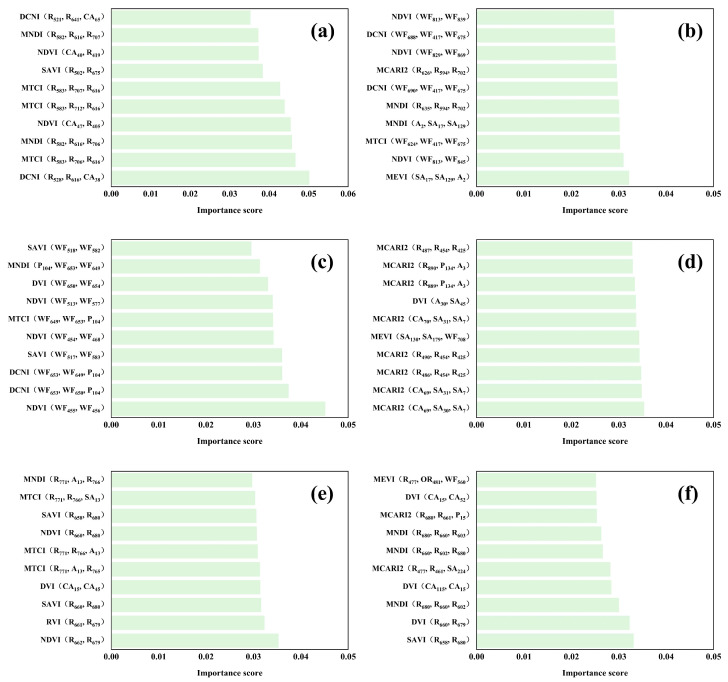
Feature importance of the top 10 spectral indices for CNN-based prediction of tea polyphenols and catechins in spring under different physical states: (**a**) tea polyphenols in field fresh leaves; (**b**) catechins in field fresh leaves; (**c**) tea polyphenols in dried tea leaves; (**d**) catechins in dried tea leaves; (**e**) tea polyphenols in tea powder; (**f**) catechins in tea powder.

To quantify the relative contributions of R, HF, and WF to CNN-based predictions, permutation importance scores of spectral indices were calculated using spring samples (Figure 6). By analyzing the feature composition of the top 10 ranked indices in each model, distinct patterns across physical states and quality parameters were observed.

For field fresh leaves, the prediction of tea polyphenols (Figure 6a) was strongly dominated by raw reflectance features. All top 10 indices contained R components, with red-edge bands (e.g., R_616_, R_641_, R_706_, R_707_) appearing repeatedly. Harmonic features, particularly cosine amplitudes (e.g., CA_40_, CA_65_, CA_38_), were involved in several indices (e.g., NDVI (CA_40_, R_419_); DCNI (R_528_, R_616_, CA_38_)), whereas WF features were absent from the top rankings. In contrast, fresh leaf catechin prediction (Figure 6b) was overwhelmingly governed by wavelet features. Most of the top-ranked indices were entirely composed of WF (e.g., NDVI (WF_813_, WF_839_); DCNI (WF_688_, WF_417_, WF_675_)), indicating a dominant contribution of multi-scale spectral information. Raw reflectance appeared only sporadically, while harmonic features (e.g., A_2_, SA_17_, SA_129_) were mainly involved through hybrid indices. For dry tea, WF features became the predominant contributors in polyphenol prediction (Figure 6c), accounting for nearly all top 10 indices (e.g., WF_518_, WF_582_, WF_650_, WF_653_), with only minor participation of harmonic phase components (e.g., P104). The overall contribution of WF exceeded 90%. In dry tea catechin models (Figure 6d), a more diversified feature composition was observed. Both reflectance-based indices (e.g., MCARI2 with R_487_, R_490_) and hybrid indices combining HF (P_134_, A_3_, CA_70_, SA_31_) and WF (e.g., WF_708_) were present. Notably, MCARI2-type indices appeared repeatedly with varying feature compositions, suggesting that the model integrates information across multiple feature domains. For powdered tea, polyphenol prediction (Figure 6e) relied primarily on the combination of higher-order harmonic features and raw reflectance. Representative indices (e.g., MNDI (R_771_, A_13_, R_766_); MTCI (R_771_, R_766_, SA_13_)) incorporated high-order amplitudes together with near-infrared reflectance (R_771_, R_766_), while red-band reflectance (e.g., R_658_, R_660_) also contributed via SAVI and NDVI. Similarly, powdered tea catechin prediction (Figure 6f) exhibited strong multi-domain fusion. Some indices were entirely derived from harmonic features (e.g., DVI (CA_15_, CA_52_)), while others combined R, HF, and WF (e.g., MEVI (R_477_, R_481_, WF_560_); MCARI2 (R_680_, R_661_, P_15_)). Harmonic components, particularly higher-order terms (e.g., CA_15_, CA_52_, P_15_, SA_224_), were frequently observed among the top-ranked features.

Overall, the dominant feature domains varied markedly across physical states. Fresh leaves relied primarily on raw reflectance and WF, dry tea was strongly dominated by WF, and powdered tea exhibited a synergistic contribution of higher-order harmonic features and raw reflectance. Additionally, distinct differences in feature dependency were observed between tea polyphenols and catechins under the same physical state.

## 3. Discussion

### 3.1. Differences in Hyperspectral Features of Tea Leaves Under Different Physical States

This study systematically revealed, via principal component analysis, the coupled effects of seasonal factors and physical states on the spectral characteristics of tea leaves. Across all seasons, the three physical states exhibited stable clustering patterns, with powdered samples forming independent clusters in spring and summer. Importantly, this persistent independence observed in our results is not merely a statistical outcome but reflects fundamental physical transformations induced by sample grinding, which directly govern light-matter interactions in hyperspectral measurements. Specifically, the stable and season-invariant clustering of tea powder observed in this study can be attributed to grinding-induced reductions in particle size and increases in specific surface area, which markedly enhance multiple scattering within the sample. Myers et al. [20] demonstrated that such particle-scale modifications systematically alter both the shape and intensity distribution of reflectance spectra, providing a mechanistic explanation for the distinct spectral space occupied by powdered samples in our PCA results. Similarly, Whatley et al. [21] reported that particle-size-related spectral effects introduced by fine grinding are highly reproducible across plant samples, which explains why tea powder in this study consistently formed independent and compact clusters in spring and summer. In tea-related research, Ouyang et al. [22] further showed that structurally homogeneous and scattering-stable powdered samples exhibit superior spectral reproducibility. These findings collectively support our observation that tea powder not only displays independent spectral clustering but also tends to achieve superior modeling performance compared with fresh and dried leaf states. In contrast, the spectral separability between field fresh leaves and dried tea leaves exhibited pronounced season-dependent behavior rather than a consistent separation pattern in this study, highlighting the complex interactions among water content, tissue structure, and biochemical composition. In spring samples, the first principal component accounted for as much as 89.4% of the total variance, indicating that spectral variability during this period was dominated by a single, strong driving factor. This result is highly consistent with the findings of Wei et al., who identified leaf water content as a primary determinant of visible–near-infrared reflectance [23]. Young spring leaves typically possess high water content and undergo rapid dehydration accompanied by cellular collapse during drying, leading to substantial and nonlinear changes in optical properties. Consequently, in spring, tea samples at different physical scales exhibited relatively higher spectral discriminability, and the observed spectral differences can be primarily attributed to water-driven structural perturbations. As the season progresses into summer and autumn, increasing leaf maturity is accompanied by structural changes such as cuticle thickening and enhanced tissue compactness. Yamashita et al. [10] reported that mature leaves exhibit lower intrinsic variability in hyperspectral reflectance, which provides a plausible explanation for the reduced spectral separability between fresh and dried leaves observed in later seasons in our results. By autumn, leaf water content is substantially reduced and tissue structure becomes more stable, resulting in smaller and more consistent spectral changes induced by processing. Under these conditions, spectral differences tend to be subtle and more difficult to resolve using low-dimensional or linear analytical approaches. In this context, the work of Luo et al. [17] is particularly relevant, as it demonstrated that exploiting the high-dimensional nature of hyperspectral data in combination with nonlinear models can effectively enhance sensitivity to complex and weak spectral variations. Therefore, consistent with our findings for autumn samples, future studies targeting late-season tea materials may benefit from adopting high-dimensional feature representations and nonlinear modeling strategies to improve discrimination and predictive performance.

### 3.2. Construction of Hyperspectral Indices Sensitive to Tea Quality Indicators Based on Iterative Computational Methods

In this study, by integrating raw reflectance spectra, harmonic features, and wavelet-derived features, and employing an iterative exhaustive search method to construct spectral indices, we successfully identified sensitive index combinations tailored to different physical states of tea leaves, fully leveraging the complementary advantages of multiple feature domains. For field fresh leaves, the optimal indices were primarily concentrated in the red-red-edge spectral region, consistent with classical understanding in vegetation biochemical monitoring. Reflectance in this region is strongly modulated by internal leaf structure and water content [24]. Accordingly, indices constructed from this region (e.g., RVI(R_583_, R_616_)) effectively capture spectral variation associated with tea polyphenols and related quality compounds, largely driven by physical state. In dried tea leaves, processing disrupts cellular structures and can induce changes in surface optical properties, potentially weakening or masking traditional reflectance peak-trough features. Here, features extracted via harmonic analysis and wavelet transform, in both frequency and localized time-frequency domains, demonstrated unique value. Wavelet transforms suppress background noise and enhance local details, while harmonic analysis separates low- and high-frequency oscillations associated with specific chemical bonds or physical properties [25,26]. When raw reflectance signals are distorted by structural changes, these frequency-domain transformations extract more robust signal patterns. The prevalence of hybrid harmonic-wavelet features (e.g., SA_130_, SA_179_, WF_708_) among the optimal indices for dried tea leaves reflects this principle, compensating for information loss due to overall signal attenuation and improving prediction stability. For tea powder, sample homogenization into fine particles causes spectra to be predominantly governed by particle-scale scattering. Myers et al. [20] reported that particle size and multiple scattering significantly influence the shape and high-frequency oscillations of powder spectra. In our study, optimal indices for tea powder were almost entirely dominated by high-order harmonic components (e.g., A_13_, R_766_, R_771_), likely because harmonic features, particularly high-frequency components, are highly sensitive to oscillations modulated by particle scattering, serving as key carriers for chemical composition prediction. The iterative and exhaustive search algorithm employed here offers the advantage of constructing customized indices for specific sample states. This method systematically evaluates all possible feature combinations and objectively selects the most representative sensitive indices, avoiding the subjectivity inherent in manual selection. This approach aligns with recent advances in hyperspectral phenotyping, such as the automated vegetation index optimization framework developed by Koh et al., which demonstrated the ability to generate adaptive indices surpassing traditional fixed indices [27]. Furthermore, the observation that three-feature indices consistently outperformed two-band indices underscores the importance of incorporating additional information dimensions to enhance spectral representation and improve model robustness.

### 3.3. Development of Tea Quality Prediction Models by Integrating Multi-Type Spectral Indices Using Deep Learning Algorithms

The CNN model consistently outperformed GPR and XGBoost across seasons and physical states (Table 3), achieving the highest prediction accuracy and stability. This aligns with Wang et al. [28], who showed that CNN-based models effectively extract hierarchical spectral features for superior tea quality prediction. The better performance of dried tea leaves and tea powder compared with field fresh leaves is due to reduced moisture interference and increased spectral homogeneity. Mao et al. [29] demonstrated that dried and ground tea samples produce more consistent spectral signals. Seasonal effects were also observed, with autumn samples showing higher accuracy than summer and spring. Huang et al. [30] noted that lower moisture in autumn stabilizes spectral–chemical relationships, explaining the improved prediction. One-way ANOVA of CNN predictions showed significant seasonal differences, with tea polyphenols and catechins following summer > autumn > spring. No significant differences were observed among the three physical states within the same season. This confirms the model’s accuracy, as it captures natural seasonal variation and provides consistent predictions across different physical states. Feature importance analysis showed that CNN adaptively uses different feature domains (Figure 6). Field fresh leaves relied on raw reflectance and wavelet features, dried tea leaves on wavelet features, and tea powder on higher-order harmonic components combined with near-infrared reflectance. Luo et al. [17] demonstrated that CNN-extracted deep features capture subtle spectral variations associated with tea polyphenol content, supporting the model’s adaptability. Different physical states also exhibit distinct spectral characteristics; dried tea leaves and tea powder are more homogeneous and stable, as highlighted by Ouyang et al. [22]. In practice, field fresh leaves are suitable for non-destructive, real-time monitoring, dried tea leaves for process control, and tea powder for laboratory calibration.

The study focused on tea polyphenols and catechins, but Mao et al. [29] showed that hyperspectral imaging can capture multiple biochemical components during tea processing. Extending the model to other quality-related compounds, such as free amino acids and caffeine, would enable a more comprehensive tea quality assessment. Gong et al. [31] reported that hyperspectral imaging combined with machine learning successfully detects tea polyphenols in Fu brick tea, suggesting feasibility across different tea cultivars and product types. Future studies incorporating multi-region and multi-cultivar datasets would enhance robustness. The proposed method allows real-time field monitoring of field fresh leaves and rapid evaluation of dried tea leaves, while tea powder, due to its homogenization and spectral stability, serves as a high-precision reference for calibration and method development. Overall, this study provides a hyperspectral-based tea quality prediction framework combining multi-domain feature extraction with deep learning.

## 4. Materials and Methods

### 4.1. Experimental Design

Hyperspectral data acquisition and tea sample collection in this study were conducted in three representative tea plantations located in Jiangsu Province, China, with the objective of identifying spectral features sensitive to key tea quality constituents and developing non-destructive monitoring models. All experimental plantations were managed using standard agronomic practices, ensuring that tea plants were not subjected to significant stress from drought, weeds, or pests during the growing period. Detailed information on sample collection is provided in Table 6.

### 4.2. Determination of Tea Leaf Quality Components

After hyperspectral sensor measurements were completed, tea samples were collected at each sampling point. Specifically, a 0.5 m × 0.5 m sampling frame was placed over the canopy of selected tea plants, and shoots consisting of one bud and one leaf were harvested within the frame. The collected samples were immediately transported to the laboratory for further processing. Field fresh leaves were first subjected to enzyme inactivation using microwave treatment, after which the samples were dried in an oven at 80 °C for 24 h until a constant weight was achieved. The dried samples were then ground using a grinder and passed through a 1 mm sieve to obtain tea powder. Finally, the powdered samples were sealed in self-locking bags and stored for subsequent chemical composition analysis.

All biochemical constituents were determined in accordance with Chinese national standard methods. Tea polyphenol content and catechins were quantified following GB/T 8313-2018 [32].

### 4.3. Hyperspectral Data Collection

#### 4.3.1. Field Hyperspectral Measurements of Fresh Tea Leaves

**Figure 7:**
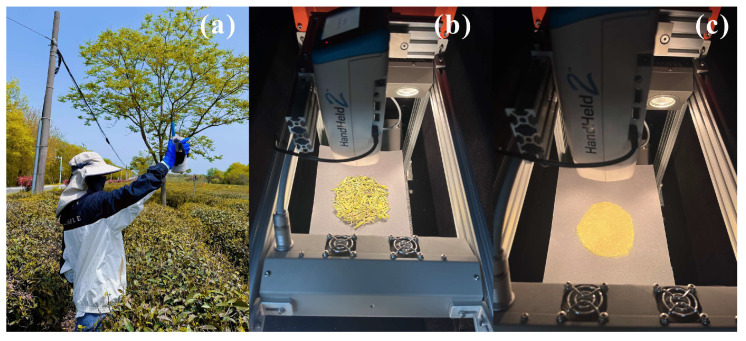
Hyperspectral measurement setups for tea samples in different physical states: (**a**) field fresh leaves under field conditions; (**b**) dried tea leaves under laboratory conditions; (**c**) tea powder under laboratory conditions.

At each sampling site, geographic coordinates were recorded using a CHCNAV K90 GNSS (Global Navigation Satellite System) device (Shanghai Huace Navigation Technology Ltd., Shanghai, China). Canopy spectral reflectance of tea plants was measured using an ASD HandHeld 2 spectroradiometer (ASD Inc., Boulder, CO, USA) (spectral range: 325–1075 nm; spectral resolution: 1 nm) (Figure 7a). Measurements were conducted under clear and calm weather conditions between 10:00 and 14:00 local time. The fiber-optic probe was held vertically at a height of 1 m above the canopy, and a white reference panel was used for calibration before and after each measurement. For each sampling point, the final reflectance spectrum was obtained by averaging three replicate measurements.

#### 4.3.2. Laboratory Hyperspectral Measurements of Dried Tea Leaves and Powdered Samples

Under laboratory conditions, hyperspectral measurements were conducted on the collected tea samples after oven-drying to constant weight to simulate the dried tea leaves state. All prepared samples were sequentially placed on a dark-box experimental platform to eliminate interference from ambient light. The measurement geometry was set with a working distance of 18 cm, a field of view of 25°, and a sample diameter of 8 cm. An ASD HandHeld 2 spectroradiometer was used to acquire spectral information from the samples (Figure 7b). Prior to data acquisition, spectral calibration was performed using a standard white reference panel to ensure measurement accuracy. Each sample was scanned three times within the dark box, and the average spectrum was taken as the reflectance value for that sample. After completion of the dried tea leaves measurements, the samples were ground into homogeneous powder using a grinder, and hyperspectral reflectance data of the tea powder were acquired using the same measurement protocol (Figure 7c).

### 4.4. Hyperspectral Data Processing

#### 4.4.1. Extraction of Harmonic Features and Wavelet Features

In this study, the original spectral reflectance measured within the wavelength range of 400−890 nm was used, as spectra in this region exhibited relatively smooth curves with low noise levels. The 891–1075 nm region was excluded due to its relatively lower signal quality under field measurement conditions, which may introduce instability into the models. First, harmonic analysis was introduced. The core concept of this method is to decompose a function into a sum of sine and cosine components, and it is commonly used for analyzing periodic signals. In this study, discrete spectral curves were treated as periodic functions and decomposed accordingly, enabling effective characterization of the spectral energy distribution pattern as well as the relationships among different wavelength bands. Specifically, a spectral curve R(k) = (r1, r2, …, rN) was decomposed as follows:

(1) $$R\left(k\right)=\frac{A_{0}}{2}+\sum_{n=1}^{\infty }\left(A_{n}\cos\frac{2\pi k}{N}+B_{n}\sin\frac{2\pi k}{N}\right)$$

The n-th harmonic expansion obtained from the analysis is represented as:

(2) $$f(k)=\frac{A_{0}}{2}+\sum_{n=1}^{\infty }C_{n}\sin\left(\frac{2\pi \mathrm{nk}}{N}+\varphi_{n}\right)$$

where A_0_/2 is the constant (average) term, C_n_ is the amplitude of the n-th harmonic, φ_n_ is the phase of the n-th harmonic.

The harmonic parameters are calculated as follows:

(3) $$A_{n}=\frac{2}{N}\sum_{k=1}^{N}r_{k}\cos\left(\frac{2\pi \mathrm{nk}}{N}\right)$$

(4) $$B_{n}=\frac{2}{N}\sum_{k=1}^{N}r_{k}\sin\left(\frac{2\pi \mathrm{nk}}{N}\right)$$

(5) $$C_{n}=\sqrt{{A_{n}}^{2}+{B_{n}}^{2}}$$

(6) $$\varphi_{n}=\tan^{-1}\left(A_{n}+B_{n}\right)$$

Here, A_n_, B_n_, C_n_ and φ_n_ denote the cosine amplitude (CA), sine amplitude (SA), resultant amplitude (A), and phase (P) of the n-th harmonic, respectively. These parameters provide key information for interpreting spectral structure: A_0_/2 represents the mean energy level of the spectrum; C_n_ characterizes the magnitude of spectral variation across wavelength bands; and φ_n_ determines the location of energy peaks. Low-order harmonic components primarily capture the overall spectral shape and dominant trends, whereas higher-order components contain finer-scale fluctuations but are more susceptible to noise. Consequently, harmonic decomposition enhances informative spectral features while suppressing noise by retaining low-order harmonics, and simultaneously achieves data compression, thereby highlighting spectrally meaningful characteristics.

Meanwhile, the CWT was employed to extract localized time-frequency characteristics of the spectra. The CWT analyzes a signal through the scaling and translation of a mother wavelet function, and its transform is defined in Equations (7) and (8).

(7) $$\varphi_{s,\lambda }\left(k\right)=\frac{1}{\surd s}\varphi \frac{(k-\lambda )}{s}$$

(8) $$w_{f}\left(s,\lambda \right)=<f,\varphi_{s,\lambda }\geq =\int_{-\infty }^{+\infty }f\left(k\right)\varphi_{s,\lambda }\left(k\right)\mathrm{dk}$$

Here, φ(k) denotes the mother wavelet function, while φ_s,λ_(k) represents the scaled and translated version of the mother wavelet. The parameter S is the decomposition scale factor, which was set to 3, 4, 5, and 6 in this study, and λ is the translation factor, expressed in terms of wavelength. The resulting wavelet coefficients, w_f_(s,λ) represent the extracted wavelet features and characterize the local properties of the spectral signal at different scales and positions, thereby enabling fine-grained analysis and feature extraction of spectral details. Compared with conventional Fourier transform methods, wavelet transform provides simultaneous information in both the wavelength and frequency domains and exhibits strong localization capability. This property makes it particularly suitable for analyzing non-stationary signals or signals with pronounced local variations. By selecting appropriate scale and translation parameters, the continuous wavelet transform can effectively capture localized spectral features associated with tea quality, thereby improving the accuracy and robustness of prediction models. In this study, the original spectral reflectance within the 400–890 nm range was subjected to continuous wavelet transform at multiple scales (s = 3, 4, 5, and 6) to examine spectral details across different frequency ranges. Ultimately, wavelet coefficients corresponding to wavelength bands showing the highest correlation with tea quality components at each scale were selected as representative wavelet features characterizing local spectral fluctuations.

#### 4.4.2. Construction of Hyperspectral Indices

To fully exploit complementary information from different feature domains and enhance monitoring accuracy, a series of hyperspectral indices were systematically constructed by integrating features derived from R, HF, and WF. Specifically, all individual features from these three domains were combined into a unified feature set, including each spectral band from R, each harmonic parameter such as CA, SA, A, and P at different orders, as well as the wavelet features derived from WF. Based on this pooled feature set, an exhaustive iterative algorithm was applied to arbitrarily select two features (λ_1_, λ_2_) from any of the three domains and substitute them into five commonly used two-band vegetation index formulations, generating the Difference Vegetation Index (DVI), Normalized Difference Vegetation Index (NDVI), Ratio Vegetation Index (RVI), Soil-Adjusted Vegetation Index (SAVI, with soil adjustment factor L = 0.5), and Modified Simple Ratio (MSR). Furthermore, to explore more complex feature combinations, three features (λ_1_, λ_2_, λ_3_) were arbitrarily selected from the pooled set to construct five types of triple-feature indices, including the Desertification Difference Index (DCNI), MERIS Terrestrial Chlorophyll Index (MTCI), Modified Normalized Difference Index (MNDI), Modified Chlorophyll Absorption in Reflectance Index 2 (MCARI2), and Modified Environment Vegetation Index (MEVI) (Table 7).

### 4.5. Data Analysis

#### 4.5.1. Dataset Definition

Based on the above formulations, dual-feature and triple-feature spectral indices were systematically generated from the fused spectral features. To construct an efficient and non-redundant feature set for model development, the large number of candidate spectral indices produced by these methods was further subjected to a rigorous screening procedure. Specifically, the coefficient of determination (R^2^) between each candidate index and each tea quality parameter was calculated. For each quality indicator, the top 10 index combinations with the highest absolute correlation were independently selected from the dual-feature and triple-feature index pools. This screening process was conducted separately for each combination of season (spring, summer, and autumn) and physical state (field fresh leaves, dried tea leaves, and tea powder), ensuring that the selected indices were optimally tailored to the specific spectral characteristics of each condition. Subsequently, 50 dual-feature indices and 50 triple-feature indices were selected from their respective pools across all conditions. These 100 indices were then used as input features for subsequent model construction, with separate models developed for each combination of season, physical state, and quality parameter. By integrating multi-domain information from original, harmonic, and wavelet features and encompassing both dual-feature and triple-feature structures, this selection strategy ensures sufficient representation of spectral variability across diverse conditions while maintaining computational feasibility, thereby enabling a comprehensive evaluation of the effectiveness and stability of multi-domain and multi-scale spectral features for non-destructive tea quality monitoring.

#### 4.5.2. Model Construction

In this study, three machine learning algorithms, XGBoost, GPR, and CNN, were employed to develop prediction models for tea quality components. XGBoost is an ensemble learning algorithm based on the gradient boosting framework, which sequentially constructs classification and regression trees (CART) and accumulates residual gradients to achieve a balance between bias and variance. The XGBoost modeling procedure consisted of the following steps: (1) Data preparation: a data matrix was constructed using the 100 selected spectral indices as independent variables and the corresponding quality contents as dependent variables, followed by spectral data cleaning; (2) Training and validation: a ten-fold cross-validation strategy was applied to divide the dataset into training and testing subsets to ensure model stability and robustness; (3) Model construction: a total of 1000 CART regression trees were sequentially built under the gradient boosting framework, with a fixed learning rate (η = 0.05), which was empirically determined based on preliminary experiments to balance model complexity and generalization ability; (4) Iterative optimization: predictions from individual trees were accumulated iteratively to minimize the squared error loss function; (5) Evaluation: predictions from the ten folds were aggregated, and the R^2^, root mean square error (RMSE), and relative error (RE) were calculated to evaluate model performance. GPR is a nonparametric regression approach grounded in Bayesian inference, in which kernel functions are used to characterize smoothness and correlation structures in the input space. The GPR modeling workflow included: (1) Data preprocessing: Z-score standardization was applied to spectral features, and target variables were normalized; (2) Dimensionality reduction: principal component analysis (PCA) was employed to reduce the spectral data to a subspace explaining 95% of the cumulative variance, thereby alleviating multicollinearity and computational burden while preserving dominant spectral information relevant to regression; (3) Kernel configuration: a composite kernel composed of a radial basis function kernel and a white noise kernel was constructed to capture nonlinear relationships and noise characteristics; (4) Parameter optimization and prediction: kernel parameters were optimized by maximizing the marginal likelihood, and both prediction and uncertainty estimation were performed within a ten-fold cross-validation framework; (5) Evaluation: after inverse transformation of predicted values, R^2^, RMSE, and RE were calculated to assess model performance. CNNs are deep learning architectures designed to process grid-structured data and are capable of automatically extracting hierarchical spectral features. To jointly exploit local spectral patterns, inter-band dependencies, and global contextual information in hyperspectral data, a CNN-BiLSTM model incorporating a multi-head attention mechanism was developed in this study. The model framework comprised: (1) Model architecture: the convolutional feature extraction module consisted of five one-dimensional convolutional layers (kernel sizes 5, 5, 3, 3, and 3) with Batch Normalization and LeakyReLU activation, combined with squeeze-and-excitation (SE) channel attention and residual connections to enhance informative spectral features. The extracted features were then fed into three stacked bidirectional long short-term memory (BiLSTM) layers (hidden size = 384) to capture long-range spectral dependencies. A multi-head self-attention layer (8 heads) was subsequently applied to adaptively weight global contextual information. (2) Regularization and initialization: dropout and early stopping were applied to mitigate overfitting, while Kaiming initialization and Xavier initialization were used for convolutional and fully connected layers, respectively, to stabilize model training. (3) Model training: the model was optimized using the SmoothL1Loss function and the AdamW optimizer, with a cosine annealing learning-rate schedule. Training was performed for up to 800 epochs within a ten-fold cross-validation framework. (4) Evaluation: the best model from each fold was retained, and aggregated predictions were used to calculate R^2^, RMSE, and relative error (RE) for performance assessment.

Model training and evaluation were conducted using a unified data partitioning strategy to ensure a fair comparison of generalization performance across all models. The entire dataset was randomly divided into ten mutually exclusive subsets following a sampling strategy without replacement, ensuring that training and validation data remained strictly independent in each iteration. During the modeling process, nine subsets were used for model training and the remaining subset was used for validation, and this procedure was repeated ten times so that each subset served as the validation set exactly once. Consequently, each sample was used once for validation and multiple times for training throughout the evaluation process. All preprocessing steps, including feature selection, standardization, and dimensionality reduction, were performed exclusively within each training subset to avoid information leakage. For each iteration, predictive performance metrics, including the coefficient of determination (R^2^), root mean square error (RMSE), and relative error (RE), were calculated independently, and the final model performance was defined as the average of the ten validation results. This approach inherently represents a rigorous form of external validation, as the validation subset in each iteration was completely excluded from the model training process, thereby ensuring strict data independence. Moreover, by repeating the procedure across ten iterations, the stability of the model under different data partitions can be comprehensively evaluated, effectively reducing the variability associated with a single random split and yielding more representative and reliable performance estimates.

(9) $$\mathrm{RMSE}=\sqrt{\frac{1}{n}\times \sum_{i=1}^{n}{\left(P_{i}-O_{i}\right)}^{2}}$$

(10) $$\mathrm{RE}\left(\%\right)=100\times \sqrt{\frac{1}{n}\times \sum_{i=1}^{n}{\left(\frac{P_{i}-O_{i}}{O_{i}}\right)}^{2}}$$

where n represents the number of samples, O_i_ and P_i_ represent the observed and predicted values, respectively.

## 5. Conclusions

This study demonstrates that integrating raw reflectance, harmonic, and wavelet-derived features can effectively improve tea quality prediction. Compared with two-feature indices, hyperspectral indices constructed from three features consistently achieved higher accuracy, with R^2^ values ranging from 0.48 to 0.71. Among the three modeling approaches compared, the convolutional neural network exhibited the best overall performance. The highest prediction accuracy was observed for autumn tea powder, where R^2^ reached 0.81 for tea polyphenols and 0.76 for catechins. Model performance also varied distinctly with season and physical state: accuracy was higher in autumn than in summer and spring, and better performance was obtained for tea powder and dried tea leaves than for field fresh leaves. Overall, the proposed framework provides a reliable approach for non-destructive tea quality evaluation.

## Figures and Tables

**Table 1:** Seasonal variations in tea quality parameters across different tea plantations.

Quality	Season	Min	Max	Mean ± SD ^1^	CV
Tea polyphenols (%)	Spring	17.09	25.70	21.54 ± 1.94 ^c^	9.02
	Summer	21.71	29.03	25.82 ± 1.97 ^a^	7.63
	Autumn	19.41	25.36	22.48 ± 1.62 ^b^	7.20
Catechins (%)	Spring	13.27	23.90	17.43 ± 2.13 ^c^	12.24
	Summer	18.04	24.50	20.08 ± 1.42 ^a^	7.07

^1^ Different superscript letters within the same quality parameter indicate significant differences among seasons (Tukey HSD, *p* < 0.05), with a > b > c. Note: SD, standard deviation; CV, coefficient of variation.

**Table 2:** Optimal two-feature and three-feature hyperspectral indices constructed in this study and their R^2^ obtained from univariate linear relationships with quality indicators.

Season	Parameter	Feature Number	Field Fresh Leaves			Dried Tea Leaves			Tea Powder		
			Index	Band Combination	R^2^	Index	Band Combination	R^2^	Index	Band Combination	R^2^
Spring	Tea polyphenols	Two	RVI	R_583_, R_616_	0.58	DVI	WF_650_, WF_653_	0.44	RVI	R_661_, R_680_	0.53
		Three	MCARI2	R_641_, R_583_, R_707_	0.65	MEVI	SA_102_, P_3_, P_8_	0.54	MNDI	A_13_, R_766_, R_771_	0.63
	catechins	Two	MSR	A_2_, SA_17_	0.54	SAVI	R_549,_ R_557_	0.35	DVI	CA_15_, CA_46_	0.40
		Three	MTCI	R_696_, R_545_, R_552_	0.59	MEVI	SA_130_, SA_179_, WF_708_	0.48	MTCI	R_736_, R_763_, R_755_	0.49
Summer	Tea polyphenols	Two	SAVI	CA_30_, CA_79_	0.35	RVI	A_114_, R_495_	0.36	RVI	R_652_, SA_200_	0.46
		Three	MCARI2	CA_17_, CA_84_, P_25_	0.48	MTCI	R_805_, R_864_, R_851_	0.54	MCARI2	R_681_, R_662_, SA_200_	0.56
	catechins	Two	SAVI	R_606_, R_620_	0.44	SAVI	CA_13_, CA_153_	0.53	DVI	CA_21,_ CA_22_	0.41
		Three	DCNI	R_617_, WF_727_, R_585_	0.56	DCNI	WF_540_, WF_464_, R_489_	0.58	DCNI	P_21_, P_22_, A_56_	0.60
Autumn	Tea polyphenols	Two	RVI	WF_510_, WF_653_	0.64	RVI	WF_772_, WF_775_	0.63	RVI	R_416_, WF_469_	0.65
		Three	MCARI2	WF_661_, WF_505_, P_78_	0.72	DCNI	WF_658_, WF_423_, CA_195_	0.72	DCNI	CA_16_, WF_848_, A_173_	0.70
	catechins	Two	DVI	R_435_, R_460_	0.47	DVI	P_2_, SA_185_	0.67	RVI	WF_401_, WF_719_	0.54
		Three	MCARI2	WF_789_, WF_778_, WF_756_	0.57	MNDI	SA_11_, R_798_, R_810_	0.71	MNDI	WF_779_, WF_705_, WF_687_	0.66

**Table 3:** Non-destructive prediction models for tea quality indicators developed by integrating multi-feature hyperspectral indices using XGBoost, GPR and CNN.

Season	Parameter	Metric	Tea Polyphenols			Catechins		
			R^2^	RMSE	RE	R^2^	RMSE	RE
Spring	Field fresh leaves	XGB	0.57	1.27	5.89	0.51	1.50	8.95
		GPR	0.63	1.17	5.57%	0.57	1.38	8.48%
		CNN	0.74	0.98	4.65%	0.72	1.11	7.00%
	Dried tea leaves	XGB	0.48	1.43	6.70	0.66	1.23	7.38
		GPR	0.57	1.26	5.97%	0.76	1.04	6.30%
		CNN	0.70	1.06	5.01%	0.75	1.05	6.45%
	Tea powder	XGB	0.62	1.19	5.53	0.53	1.45	8.36
		GPR	0.69	1.07	4.99%	0.60	1.34	8.08%
		CNN	0.81	0.85	3.93%	0.75	1.07	6.42%
Summer	Field fresh leaves	XGB	0.66	0.95	3.77	0.61	0.90	4.42
		GPR	0.75	0.80	3.15%	0.72	0.74	3.66%
		CNN	0.71	0.86	3.39%	0.81	0.62	3.03%
	Dried tea leaves	XGB	0.58	1.04	4.09	0.69	0.81	4.01
		GPR	0.62	0.98	3.85%	0.79	0.64	3.16%
		CNN	0.72	0.84	3.25%	0.77	0.68	3.43%
	Tea powder	XGB	0.63	0.97	3.83	0.73	0.74	3.56
		GPR	0.58	1.03	4.07%	0.74	0.71	3.56%
		CNN	0.75	0.80	3.16%	0.82	0.60	3.00%
Autumn	Field fresh leaves	XGB	0.66	0.93	4.24	0.52	1.26	6.92
		GPR	0.70	0.87	3.98%	0.67	1.03	5.72%
		CNN	0.77	0.77	3.51%	0.75	0.90	4.86%
	Dried tea leaves	XGB	0.63	0.99	4.48	0.68	1.02	5.60
		GPR	0.71	0.87	3.96%	0.70	0.98	5.40%
		CNN	0.79	0.74	3.37%	0.84	0.73	4.15%
	Tea powder	XGB	0.65	0.96	4.27	0.61	1.13	6.26
		GPR	0.70	0.87	3.93%	0.68	1.02	5.67%
		CNN	0.81	0.70	3.11%	0.76	0.89	5.03%

**Table 4:** Seasonal differences in tea quality parameters within each physical state.

Quality Parameter	Physical State	Season	Mean (%) ± SD	F	*p*	Tukey’s HSD
Tea polyphenols	Field fresh leaves	Spring	21.54 ± 1.68	124.90	<0.001	spring < autumn < summer
		Summer	25.83 ± 1.29			
		Autumn	22.52 ± 1.34			
	Dried tea leaves	Spring	21.45 ± 1.49	140.03	<0.001	spring < autumn < summer
		Summer	25.79 ± 1.30			
		Autumn	22.50 ± 1.38			
	Tea powder	Spring	21.55 ± 1.71	113.72	<0.001	spring < autumn < summer
		Summer	25.80 ± 1.39			
		Autumn	22.44 ± 1.40			
Catechins	Field fresh leaves	Spring	17.51 ± 1.79	37.12	<0.001	spring < autumn < summer
		Summer	20.01 ± 1.20			
		Autumn	19.00 ± 1.53			
	Dried tea leaves	Spring	17.45 ± 1.76	40.35	<0.001	spring < autumn < summer
		Summer	20.11 ± 1.26			
		Autumn	19.11 ± 1.70			
	Tea powder	Spring	17.49 ± 1.68	46.50	<0.001	spring < autumn < summer
		Summer	20.16 ± 1.26			
		Autumn	19.04 ± 1.39			

**Table 5:** Differences among physical states within each season.

Season	Quality Parameter	Physical State	Mean (%) ± SD	F	*p*	Tukey’s HSD
Spring	Tea polyphenols	Field fresh leaves	21.54 ± 1.68	0.084	0.919	no significant differences
		Dried tea leaves	21.45 ± 1.49			
		Tea powder	21.55 ± 1.71			
	Catechins	Field fresh leaves	17.51 ± 1.79	0.028	0.973	no significant differences
		Dried tea leaves	17.45 ± 1.76			
		Tea powder	17.49 ± 1.68			
Summer	Tea polyphenols	Field fresh leaves	25.83 ± 1.29	0.016	0.984	no significant differences
		Dried tea leaves	25.79 ± 1.30			
		Tea powder	25.80 ± 1.39			
	Catechins	Field fresh leaves	20.01 ± 1.20	0.169	0.845	no significant differences
		Dried tea leaves	20.11 ± 1.26			
		Tea powder	20.16 ± 1.26			
Autumn	Tea polyphenols	Field fresh leaves	22.52 ± 1.34	0.046	0.955	no significant differences
		Dried tea leaves	22.50 ± 1.38			
		Tea powder	22.44 ± 1.40			
	Catechins	Field fresh leaves	19.00 ± 1.53	0.065	0.937	no significant differences
		Dried tea leaves	19.11 ± 1.70			
		Tea powder	19.04 ± 1.39			

**Table 6:** Basic information on the tea plant sampling experiment conducted in 2024.

Experiment No.	Tea Garden Location	Cultivar	Number of Sampling Points	Sampling Stage
1	Pingshan (32.46° N, 118.84° E)	Jiukeng	96	15 April; 1 May; 5 July; 22 September
2	Juyuanchun (32.33° N, 119.05° E)	Longjingchangye Jiukeng	36	18 April; 4 July; 28 September
3	Tianwangwu (31.13° N, 120.28° E)	Chuyeqi	36	8 May; 31 July; 29 September

**Table 7:** Construction of dual-feature and triple-feature spectral indices.

Index Type	Index Name	Formula	Reference
Dual-feature indices	Difference Vegetation Index (DVI)	λ_1_ − λ_2_	[33]
	Normalized Difference Vegetation Index (NDVI)	(λ_1_ − λ_2_)/(λ_1_ + λ_2_)	[34]
	Ratio Vegetation Index (RVI)	λ_1_/λ_2_	[35]
	Soil-Adjusted Vegetation Index (SAVI)	((λ_1_ − λ_2_)(1 + L))/(λ_1_ + λ_2_ + L)	[36]
	Modified Simple Ratio (MSR)	(λ_1_/λ_2_ − 1)/(λ_1_/λ_2_ + 1)^0.5^	[37]
Triple-feature indices	Desertification Difference Index (DCNI)	((λ_1_ − λ_2_)/(λ_2_ − λ_3_))/(λ_1_ − λ_3_ + 0.03)	[38]
	MERIS terrestrial chlorophyll index (MTCI)	(λ_1_ − λ_2_)/(λ_2_ − λ_3_)	[39]
	Modified Normalized Difference Index (MNDI)	(λ_1_ − λ_2_)/((λ_1_ − λ_2_) − (λ_2_ − λ_3_))	[40]
	Modified chlorophyll absorption in reflectance index2 (MCARI2)	((λ_1_ − λ_2_) − 0.2(λ_1_ − λ_3_))(λ_1_/λ_2_)	[41]
	Modified Environment Vegetation Index (MEVI)	(λ_1_ − λ_2_)/(λ_1_ + 2.5λ_2_ − 1.5λ_3_ + 1)	[42]

## Data Availability

All data and materials are available upon request.
